# Global burden of thyroid cancer among adolescents and young adults, 1990-2021, and projections to 2050: an analysis based on the GBD 2021

**DOI:** 10.3389/fendo.2025.1503144

**Published:** 2025-04-14

**Authors:** Ming-Jie Jia, Shen Wang, Yu Li, Xing-Ning Liu, Feng Jiang, Hui-Lin Li

**Affiliations:** ^1^ Ruikang Hospital Affiliated to Guangxi University of Chinese Medicine, Nanning, Guangxi Zhuang Autonomous Region, China; ^2^ Department of Health Toxicology, School of Public Health, China Medical University (Shenbei Campus), Shenyang, Liaoning, China; ^3^ The Fourth Clinical Medical College of Guangzhou University of Chinese Medicine, Shenzhen, China; ^4^ Department of Endocrinology, Shenzhen Traditional Chinese Medicine Hospital, Shenzhen, China

**Keywords:** thyroid cancer, AYA population, GBD, ARIMA prediction, burden of disease

## Abstract

**Objective:**

To evaluate the global burden of thyroid cancer (TC) among adolescents and young adults (AYA) aged 15-39 years from 1990 to 2021, with projections to 2050, and identify demographic and regional disparities.

**Methods:**

Using Global Burden of Disease (GBD) 2021 data, we analyzed incidence, prevalence, mortality, and disability-adjusted life years (DALYs) across 204 countries. Time-series projections to 2050 were generated using autoregressive integrated moving average (ARIMA) models.

**Results:**

Global thyroid cancer incidence among AYA increased by 150% from 19,268 cases in 1990 to 48,203 in 2021, with persistent gender disparities: females exhibited a 2021 incidence rate of 2.38 per 100,000, threefold higher than males (0.88 per 100,000). Regional analysis revealed the highest burden in the Middle East and North Africa (2.49 per 100,000 in 2021). Projections indicate that by 2050, global prevalence will reach 103.62 per million, accompanied by an incidence rate of 11.41 per 100,000 and a DALYs burden of 34.41 per million, reflecting an 18% increase from 2021. Mortality rates show a modest rise from 0.37 per million in 1990 to a projected 0.42 per million in 2050. Socioeconomic disparities are pronounced: lower-Sociodemographic Index (SDI) regions face a projected 23% incidence increase by 2050, contrasting with a 12% decline in high-SDI regions, highlighting widening healthcare inequities.

**Conclusion:**

The growing burden of thyroid cancer among AYA populations demonstrates critical gender and geographic disparities, disproportionately affecting females and lower-resource regions. Mitigation requires enhanced early detection protocols, optimized treatment pathways, and targeted resource allocation to vulnerable populations.

## 1 Introduction

The global burden of thyroid cancer (TC) is rising, with significant economic and clinical impacts (1, 2). In the United States, out-of-pocket costs for TC diagnosis and treatment range from $1,425 to $17,000, with 16 to 50 per cent of patients facing financial hardship (3). The cost of papillary TC in the United States alone is projected to reach $21 billion by 2019, yet research funding remains low (4). Thyroid cancer patients have the highest rate of bankruptcy after diagnosis, at 9.3 cases per 1,000 person-years (5). In China, new cases have increased from 10,030 in 1990 to 39,080 in 2019 and are expected to reach 47,820 by 2039 (6). The rapid rise in incidence, especially in low- and middle-income countries, coupled with high diagnostic costs, exacerbates economic pressures (2). Globally, thyroid cancer survivors face both economic and psychological hardship, highlighting the urgent need for more cost-effective management and increased research funding.

TC is influenced by multiple risk factors, including genetic predisposition, exposure to radiation, and endocrine disruption, and is therefore a multifactorial disease with a significant impact on morbidity, mortality, and disability-adjusted life years (DALYs) (7). Genetic factors such as BRAF and RET/PTC mutations are associated with more aggressive forms of TC, often resulting in higher morbidity due to the need for extensive treatments and prolonged care (8, 9). Environmental factors, particularly radiation exposure during childhood, are linked to more invasive and potentially lethal types of TC, thereby increasing the risk of mortality (10). Additionally, elevated thyroid-stimulating hormone (TSH) levels have been correlated with a heightened risk of papillary thyroid carcinoma, further contributing to the disease burden (11). These risk factors cumulatively impact DALYs by increasing the years lived with disability, as patients often require lifelong monitoring and treatment for complications such as hypothyroidism, significantly affecting both their quality of life and long-term health outcomes (12).

In 2021, the global incidence, mortality, and DALYs for TC were 249,538, 44,799, and 646,741, respectively, showing an increase from 233,846 new cases and 45,575 deaths in 2019 (1, 13). This highlights the growing burden of TC worldwide. Several studies have identified long-term trends in cancer incidence and mortality at both global and regional levels. Given the significant changes in risk factors, understanding future trends is essential for adapting health systems to meet these evolving challenges. However, much of the previous research has been retrospective, focusing on overall populations rather than specific age groups. Studying TC in adolescents and young adults (AYA), aged 15-39, is particularly crucial because this group exhibits distinct patterns of disease presentation and outcomes compared to other age groups (14). The incidence of TC in AYA patients is significantly higher than in children, with a 10-fold increase observed in adolescents (15). In fact, TC is among the top five cancers diagnosed in individuals aged 15-39, emphasizing the importance of focused research on this specific age group (16).

Our study will use the Global Burden of Disease (GBD) database to explore male-female differences in TC incidence and mortality in this age group, revealing gender-specific risk factors and outcomes. In addition, we will use autoregressive integrated moving average (ARIMA) models to predict future trends in TC up to 2050.

## 2 Materials and methods

### 2.1 Data sources

The data for this study were sourced from the GBD 2021 database, which provides comprehensive epidemiological information on the incidence, prevalence, mortality, and DALYs for various diseases and risk factors across 204 countries and territories (17). TC was defined using the International Classification of Diseases (ICD-10) code C73, which includes all subtypes, such as papillary, follicular, medullary, and anaplastic carcinoma. Data on thyroid cancer from 1990 to 2021, with projections to 2050, were analysed using a cross-sectional design based on retrospective data to assess global time trends. The study examined incidence, prevalence, mortality, and DALYs, with DALYs defined as the sum of years of life lost due to premature death (YLL) and years lived with disability (YLD). To investigate the influence of socio-economic factors, the data were stratified by the Socio-Demographic Index (SDI), a composite measure of social and economic development, categorizing countries into five SDI levels: low, medium-low, medium, medium-high, and high (18). Age-standardized indicators were used to ensure comparability across regions, focusing on individuals aged 15-39, further divided into five-year subgroups. Additionally, the data were stratified by gender to assess differences in the burden of thyroid cancer between males and females.

The indicator of estimated annual percentage change (EAPC) is a summary and widely used measure of mortality trends over a specific interval, calculated from a regression model fitted to the natural logarithm of mortality, i.e. ln (rate) = α + β × (calendar year) + ϵ, with the EAPC defined as 100 × (exp (β)-1). Its 95% confidence interval (95% CI) is also obtained from the linear regression model (19).

### 2.2 Predicting thyroid cancer burden beyond 2019

In this study, autoregressive integrated moving average (ARIMA) model pairs of time series models were used to forecast future trends in incidence, prevalence, mortality, and DALYs. The ARIMA (p, d, q) model consists of three components: p represents the autoregressive term reflecting the relationship between current and past values; d is the number of differences applied to the data to make them stationary; and q is the moving average term used to smooth out random fluctuations (20). ARIMA’s ability to handle complex data patterns such as the seasonal variations commonly found in cancer incidence data has led to its widespread use in medicine, e.g. ARIMA is used in the USA to predict seasonal variations in thyroid cancer (21). It is particularly useful for long-term forecasting as it captures underlying trends that may not be immediately apparent. For example, ARIMA has been used effectively in studies to predict cancer incidence based on population data, such as analysing Australian prostate cancer data from 1982 to 2012 to predict annual values from 2014 to 2022 (22). An ARIMA model model were constructed using the R programming language to predict the burden of breast cancer from 2022 to 2050.

### 2.3 Statistical analysis

All estimates were accompanied by 95% uncertainty intervals (95% UI), calculated from the 2nd and 97.5th percentiles of 1,000 samples drawn from uncertainty distributions. The data analysis and trend estimation were performed using R software (Institute for Statistics and Mathematics) and STATA (Stata Corp LLC). Statistical significance was determined with a p-value threshold of less than 0.05.

## 3 Result

### 3.1 Disease burden in 2021

**Figure 1:**
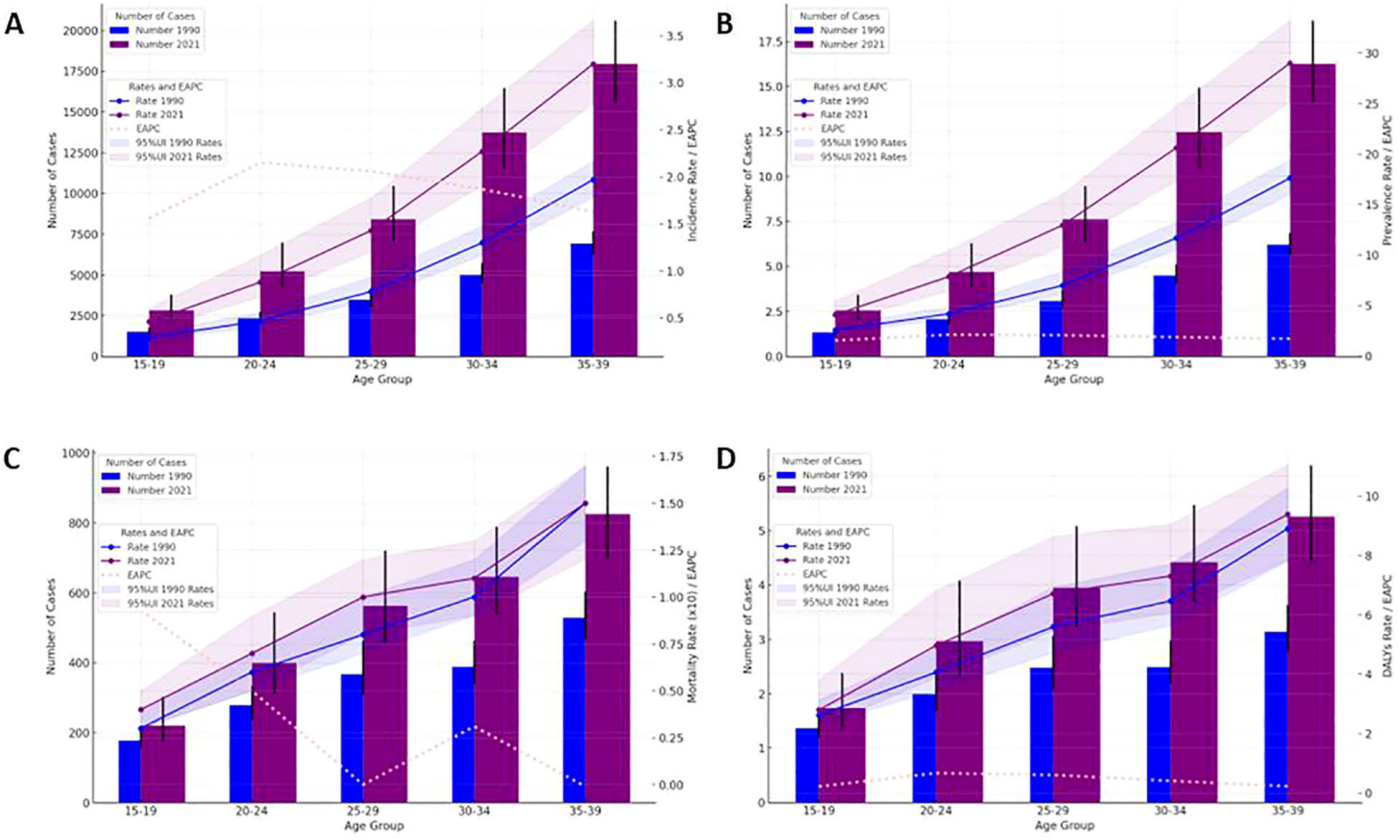
Thyroid cancer case numbers and incidence rates **(A)**, prevalence rate **(B)**, mortality **(C)** and DALYs rate **(D)** by age group (1990 & 2021) with 95% UI.

In 2021, there were 48,203 newly diagnosed TC cases, corresponding to an age-standardized incidence rate of 1.62 per 100,000 (**Table 1**). The total number of current patients was 436,144, with an age-standardized prevalence rate of approximately 14 patients per million population. There were 2,652 deaths, resulting in an age-standardized mortality rate of 0.09 per 100,000. Females exhibited higher age-at-onset morbidity, mortality, and DALY (disability-adjusted life year) rates than males. However, the estimated annual percentage change (EAPC) for all four rate values in males exceeded those in females. The age-standardized mortality rate showed a decline in regions with high-middle SDI, high SDI, and low SDI (**Tables 2**, **3**). A total of 183,485 DALYs were recorded, yielding an age-standardized DALY rate of 6.17 per 100,000 population. DALYs were most prevalent in regions with low-moderate SDI and peaked in areas with low SDI (**Table 4**). All four indicators—incidence, prevalence, mortality, and DALYs—increased with age, with EAPC decreasing as age increased. The highest EAPC was observed in the 20-24 years age group (**Figure 1**).

**Table 1:** Age-standardized and age-specific incidence rate (per 100 000) and number of cases of thyroid cancer by sex, national development index, 1990 and 2021.

	Case number		Incidence rate		EAPC (%)
	1990 (95%UI)	2021 (95%UI)	1990 (95%UI)	2021 (95%UI)	1990-2021 (95%UI)
**Global**	19268 (17499-21851)	48203 (40896-56747)	0.88 (0.8-1)	1.62 (1.37-1.91)	2.08 (1.98-2.17)
Gender					
Female	14580 (12692-16860)	34940 (28623-43505)	1.35 (1.17-1.56)	2.38 (1.95-2.97)	1.9 (1.79-2.01)
Male	4688 (4382-5130)	13264 (11196-14867)	0.42 (0.4-0.46)	0.88 (0.74-0.98)	2.62 (2.52-2.71)
SDI region					
High-middle SDI	4451 (3991-4874)	7879 (6951-9359)	0.98 (0.88-1.08)	1.79 (1.58-2.13)	2.19 (1.96-2.42)
High SDI	5654 (5421-5921)	8502 (7935-9241)	1.63 (1.56-1.71)	2.41 (2.25-2.62)	1.49 (1.27-1.72)
Low-middle SDI	2878 (2348-3871)	10741 (8540-14519)	0.63 (0.52-0.85)	1.34 (1.06-1.81)	2.4 (2.32-2.47)
Low SDI	1289 (986-1626)	5206 (3927-7512)	0.7 (0.53-0.88)	1.16 (0.87-1.67)	1.56 (1.5-1.62)
Middle SDI	4975 (4244-5866)	15844 (12557-18493)	0.66 (0.56-0.78)	1.71 (1.35-1.99)	3.15 (3.09-3.2)
Age					
15-19 years	1514 (1346-1780)	2848 (2301-3808)	0.29 (0.26-0.34)	0.46 (0.37-0.61)	1.56% (1.19-1.97)
20-24 years	2337 (2040-2734)	5231 (4272-6995)	0.47 (0.41-0.56)	0.88 (0.72-1.17)	2.15% (1.89-2.49)
25-29 years	3469 (3055-4168)	8424 (7047-10459)	0.78 (0.69-0.94)	1.43 (1.2-1.78)	2.06% (1.86-2.15)
30-34 years	5022 (4526-5718)	13747 (11566-16484)	1.3 (1.17-1.48)	2.27 (1.91-2.73)	1.87% (1.64-2.07)
35-39 years	6926 (6278-7658)	17954 (15591-20607)	1.97 (1.78-2.17)	3.2 (2.78-3.67)	1.63% (1.50-1.76)

**Table 2:** Age-standardized and age-specific prevalence rate (per 100 000) and number of cases of thyroid cancer by sex, national development index, 1990 and 2021.

	Case number (10^4)		Prevalence rate		EAPC
	1990 (95%UI)	2021 (95%UI)	1990 (95%UI)	2021 (95%UI)	1990-2021 (95%UI)
**Global**	17.23 (15.65-19.52)	43.61 (37.01-51.28)	7.86 (7.14-8.91)	14.66 (12.44-17.24)	2.12 (2.03-2.22)
Gender					
Female	13.10 (11.42-15.13)	31.71 (26.00-39.48)	12.09 (10.54-13.96)	21.64 (17.75-26.95)	1.94 (1.83-2.05)
Male	4.13 (3.86-4.51)	11.90 (10.05-13.34)	3.73 (3.48-4.07)	7.89 (6.66-8.84)	2.69 (2.59-2.78)
SDI region					
High-middle SDI	4.02 (3.60-4.40)	7.18 (6.34-8.54)	8.87 (7.96-9.72)	16.32 (14.39-19.4)	2.23 (1.99-2.46)
High SDI	5.15 (4.94-5.40)	7.77 (7.26-8.45)	14.84 (14.23-15.55)	22.01 (20.54-23.93)	1.5 (1.28-1.73)
Low-middle SDI	2.51 (2.05-3.38)	9.62 (7.65-13.02)	5.54 (4.51-7.45)	11.99 (9.53-16.23)	2.49 (2.42-2.56)
Low SDI	1.10 (0.84-1.40)	4.63 (3.49-6.70)	5.98 (4.57-7.58)	10.31 (7.78-14.92)	1.71 (1.65-1.77)
Middle SDI	4.43 (3.77-5.23)	14.38 (11.39-16.79)	5.88 (5.01-6.94)	15.5 (12.28-18.1)	3.21 (3.16-3.27)
Age					
15-19 years	1.34 (1.19-1.58)	2.57 (2.07-3.43)	2.59 (2.3-3.04)	4.11 (3.32-5.49)	1.56% (1.24-2.00)
20-24 years	2.07 (1.80-2.41)	4.70 (3.85-6.29)	4.2 (3.67-4.9)	7.88 (6.44-10.53)	2.13% (1.87-2.57)
25-29 years	3.10 (2.74-3.72)	7.63 (6.38-9.47)	7.01 (6.18-8.41)	12.96 (10.84-16.09)	2.08% (1.88-2.18)
30-34 years	4.51 (4.06-5.12)	12.46 (10.48-14.95)	11.69 (10.55-13.29)	20.61 (17.34-24.73)	1.90% (1.66-2.09)
35-39 years	6.21 (5.63-6.86)	16.26 (14.12-18.66)	17.62 (15.99-19.47)	28.99 (25.17-33.27)	1.73 (1.63-1.82)

**Table 3:** Age-standardized and age-specific mortality (per 100 000) and number of cases of thyroid cancer by sex, national development index, 1990 and 2021.

	Case number		mortality		EAPC
	1990 (95%UI)	2021 (95%UI)	1990 (95%UI)	2021 (95%UI)	1990-2021 (95%UI)
**Global**	1738 (1521-2049)	2652 (2212-3251)	0.08 (0.07-0.09)	0.09 (0.07-0.11)	0.35 (0.31-0.39)
Gender					
Female	1166 (963-1457)	1686 (1331-2243)	0.11 (0.09-0.13)	0.12 (0.09-0.15)	0.11 (0.05-0.18)
Male	572 (521-656)	966 (777-1124)	0.05 (0.05-0.06)	0.06 (0.05-0.07)	0.81 (0.77-0.85)
SDI region					
High-middle SDI	298 (260-328)	223 (198-255)	0.07 (0.06-0.07)	0.05 (0.04-0.06)	-0.9 (-1–0.8)
High SDI	185 (178-193)	143 (133-157)	0.05 (0.05-0.06)	0.04 (0.04-0.04)	-0.72 (-0.8–0.63)
Low-middle SDI	462 (380-613)	951 (761-1259)	0.1 (0.08-0.14)	0.12 (0.09-0.16)	0.46 (0.34-0.59)
Low SDI	267 (207-331)	605 (457-859)	0.14 (0.11-0.18)	0.13 (0.1-0.19)	-0.36 (-0.41–0.3)
Middle SDI	525 (455-610)	728 (604-820)	0.07 (0.06-0.08)	0.08 (0.07-0.09)	0.39 (0.36-0.43)
Age					
15-19 years	177 (156-214)	220 (173-302)	0.03 (0.03-0.04)	0.04 (0.03-0.05)	0.93 (0.54-1.32)
20-24 years	278 (236-332)	399 (312-544)	0.06 (0.05-0.07)	0.07 (0.05-0.09)	0.50 (0.25-0.75)
25-29 years	367 (310-461)	562 (456-720)	0.08 (0.07-0.1)	0.1 (0.08-0.12)	0.00 (-0.32-0.30)
30-34 years	388 (338-462)	645 (537-788)	0.1 (0.09-0.12)	0.11 (0.09-0.13)	0.31 (0.02-0.61)
35-39 years	529 (466-603)	825 (701-961)	0.15 (0.13-0.17)	0.15 (0.12-0.17)	-0.01 (-0.07-0.05)

**Table 4:** Age-standardized and age-specific DALYs rate (per 100 000) and number of cases of thyroid cancer by sex, national development index, 1990 and 2021.

	Case number (10^4)		DALYs rate		EAPC
	1990 (95%UI)	2021 (95%UI)	1990 (95%UI)	2021 (95%UI)	1990-2021 (95%UI)
**Global**	11.47 (9.97-13.46)	18.35 (15.15-22.86)	5.23 (4.55-6.14)	6.17 (5.09-7.68)	0.52 (0.48-0.56)
Gender					
Female	7.78 (6.35-9.66)	11.93 (9.47-15.92)	7.19 (5.86-8.92)	8.14 (6.47-10.87)	0.32 (0.26-0.39)
Male	3.69 (3.35-4.20)	6.42 (5.11-7.43)	3.33 (3.03-3.79)	4.25 (3.39-4.92)	0.92 (0.88-0.97)
SDI region					
High-middle SDI	1.99 (1.74-2.23)	1.69 (1.46-2.00)	4.4 (3.85-4.93)	3.84 (3.31-4.54)	-0.41 (-0.53–0.3)
High SDI	1.37 (1.27-1.51)	1.26 (1.09-1.46)	3.96 (3.66-4.34)	3.56 (3.09-4.14)	-0.12 (-0.21–0.02)
Low-middle SDI	2.98 (2.47-3.96)	6.30 (4.99-8.51)	6.57 (5.44-8.73)	7.85 (6.22-10.6)	0.54 (0.42-0.67)
Low SDI	1.71 (1.33-2.12)	3.99 (3.00-5.74)	9.27 (7.23-11.52)	8.88 (6.68-12.79)	-0.28 (-0.34–0.22)
Middle SDI	3.41 (2.95-3.95)	5.10 (4.20-5.86)	4.53 (3.92-5.24)	5.5 (4.53-6.31)	0.65 (0.61-0.69)
Age					
15-19 years	1.369 (1.20-1.63)	1.74 (1.38-2.39)	2.62 (2.31-3.14)	2.8 (2.22-3.82)	0.22 (-0.13-0.66)
20-24 years	2.00 (1.69-2.38)	2.97 (2.32-4.08)	4.07 (3.44-4.85)	4.97 (3.89-6.83)	0.67 (0.41-1.14)
25-29 years	2.48 (2.094-3.07)	3.95 (3.22-5.09)	5.6 (4.73-6.94)	6.72 (5.47-8.65)	0.61 (0.48-0.73)
30-34 years	2.49 (2.18-2.98)	4.42 (3.67-5.47)	6.47 (5.65-7.72)	7.31 (6.07-9.06)	0.41 (0.24-0.54)
35-39 years	3.1400 (2.75-3.63)	5.26 (4.40-6.20)	8.91 (7.82-10.29)	9.39 (7.84-11.06)	0.23 (0.17-0.29)

### 3.2 Regional variations in the global burden of thyroid cancer

**Figure 2:**
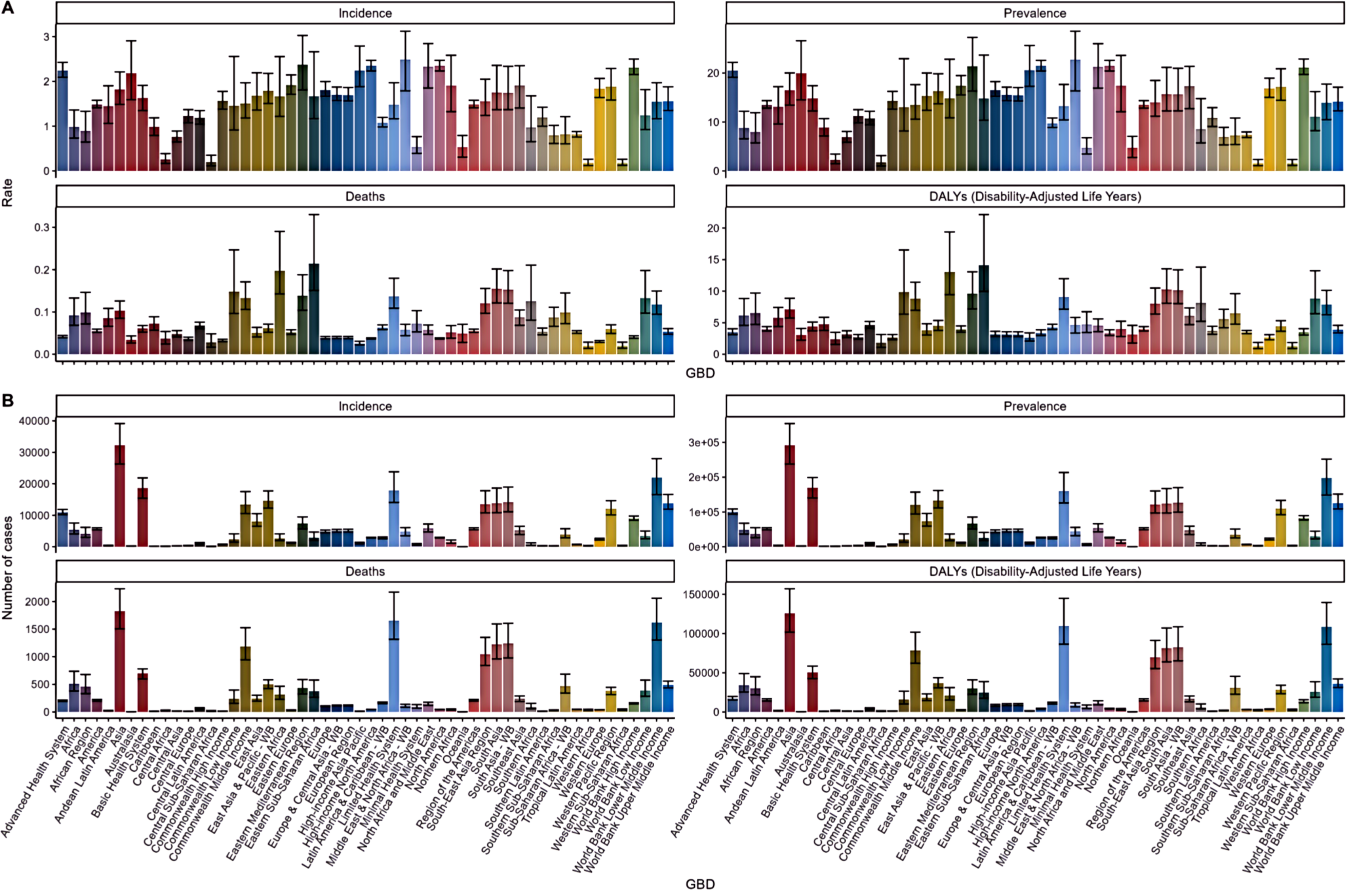
Incidence, prevalence, death and disability adjusted life year (DALY) rates and numbers of thyroid cancer disease incidence, prevalence, death and disability adjusted life year (DALY) rates **(A)** and numbers **(B)** in the AYA population and 95% UI.

The GBD database reveals substantial regional variations in the burden of TC among AYA populations. In the Middle East and North Africa (MENA), the highest incidence and prevalence rates were observed, with 2.49 (95% UI: 1.79-3.12) per 100,000 and 22.74 (95% UI: 16.34-28.53) per 100,000, respectively. MENA also exhibited the highest EPAC at 3.96 (95% UI: 3.74-4.17), while Central Europe had the lowest EPAC at -0.55 (95% UI: -0.86 to -0.25), followed by Central Asia with -0.22 (95% UI: -1.05 to 0.62). North America recorded similarly high incidence and prevalence rates, at 2.35 (95% UI: 2.23-2.47) and 21.48 (95% UI: 20.41-22.59) per 100,000, respectively. Asia dominated in total new and cumulative cases, with East Asia and the Pacific reporting 14,573 new cases and 13,267,673 cumulative cases in 2021, while South Asia followed closely with 14,177 new cases and 132,673 cumulative cases. In contrast, Western Sub-Saharan Africa had the lowest incidence and prevalence rates. As shown in **Figure 2**.

Mortality patterns also varied, with Eastern Sub-Saharan Africa reporting the highest TC mortality rate at 0.21 (95% CI: 0.15-0.33) and Western Sub-Saharan Africa showing the lowest at 0.02 (95% CI: 0.01-0.03). Asia accounted for 1,826 TC-related deaths, with South Asia contributing 1,226 of these. Europe saw notable decreases in mortality, particularly in Central and Western Europe, with EAPC of -3.15 (95% CI: -3.59 to -2.71) and -2.24 (95% CI: -2.45 to -2.03), respectively, while the Eastern Mediterranean Region and Southern Africa exhibited increasing trends with an EAPC of 1.24. Furthermore, Eastern Sub-Saharan Africa had the highest DALY rate at 14.11 (95% CI: 9.96-22.14) per 100,000, followed by South Asia at 10.29 (95% CI: 8.08-13.54), with Central Africa reporting the lowest DALY rate at 2.39 (95% CI: 1.56-3.49). Asia contributed the highest number of total DALYs, with South Asia leading at 81,363 (95% CI: 63,915-107,109), while Central Europe and Central Asia experienced the most significant changes in EPAC. It is important to note that these EAPC values reflect trends over the period from 1990 to 2021 (**Table 2**). As shown in **Figure 2**.

### 3.3 National-level burden

**Figure 3:**
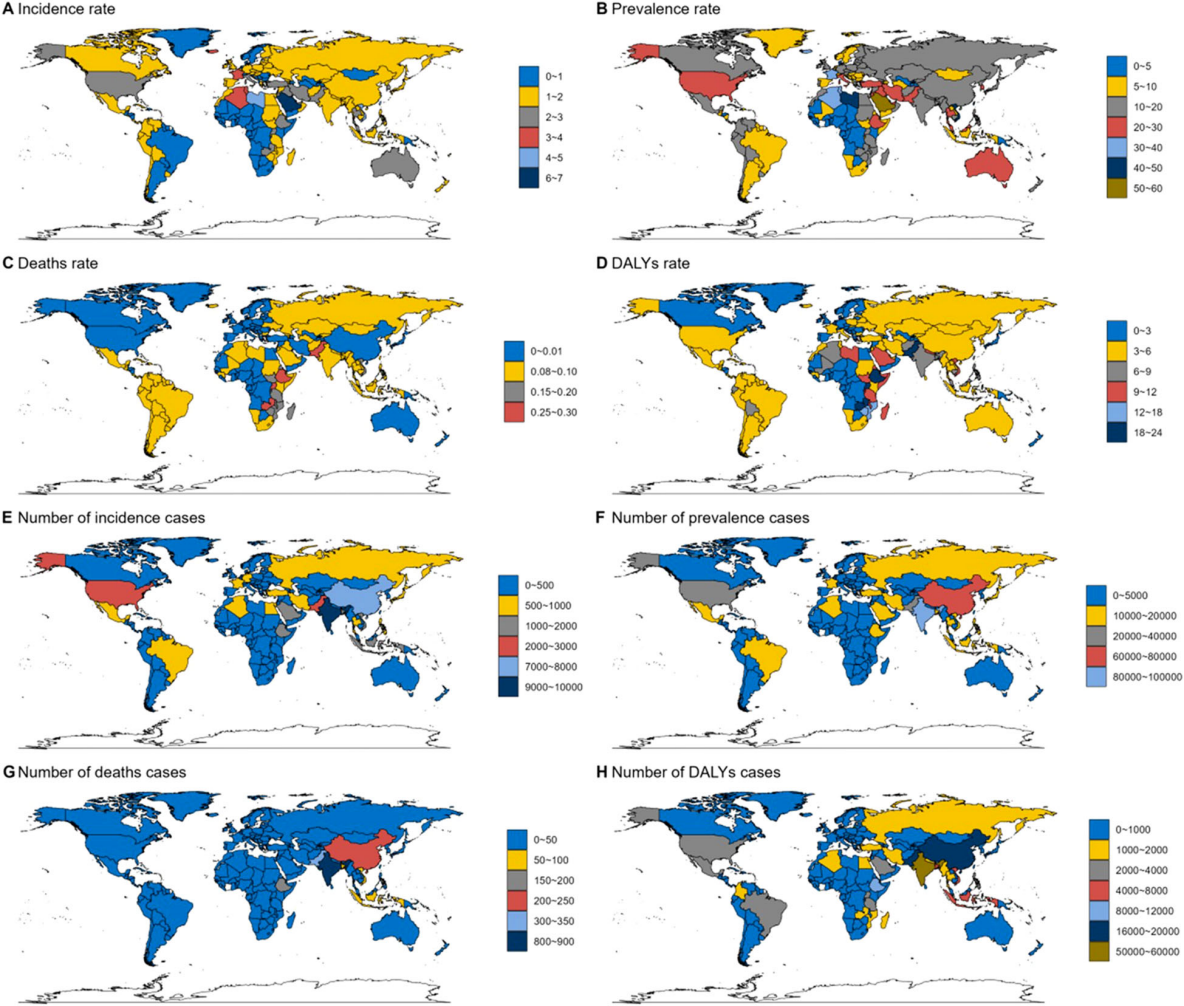
Global thyroid cancer incidence by country in 2021. **(A)** Incidence rate. **(B)** Prevalence rate. **(C)** Deaths rate. **(D)** DALYs rate. **(E)** Number of incidence cases. **(F)** Number of prevalence cases. **(G)** Number of deaths cases. **(H)** Number of DALYs cases.

In the AYA 2021 TC population, distinct regional variations in disease burden were observed. Libya, in Northern Africa, recorded the highest global DALY rate, while countries in Western Europe showed a significantly higher negative EAPC for DALY rates compared to other regions. In Sub-Saharan Africa, Ethiopia had the highest mortality rate at 0.33 (0.22-0.57) per 100,000. In contrast, developed countries such as Switzerland and Singapore reported the lowest mortality rates at 0.02 (0.01-0.02) per 100,000. South Asia, particularly India, registered the highest number of deaths (815), as well as significant counts in deaths and morbidity, with 87,637 and 9,749, respectively. In terms of prevalence, Libya, Viet Nam (Southeast Asia), and Taiwan (East Asia) exhibited the highest rates, all exceeding 40 per million, with Libya’s figures standing at 5.04 (2.75-7.75), Viet Nam at 4.66 (2.16-7.08), and Taiwan at 4.55 (3.6-5.65) (**Figure 3**).

### 3.4 Cluster analysis

**Figure 4:**
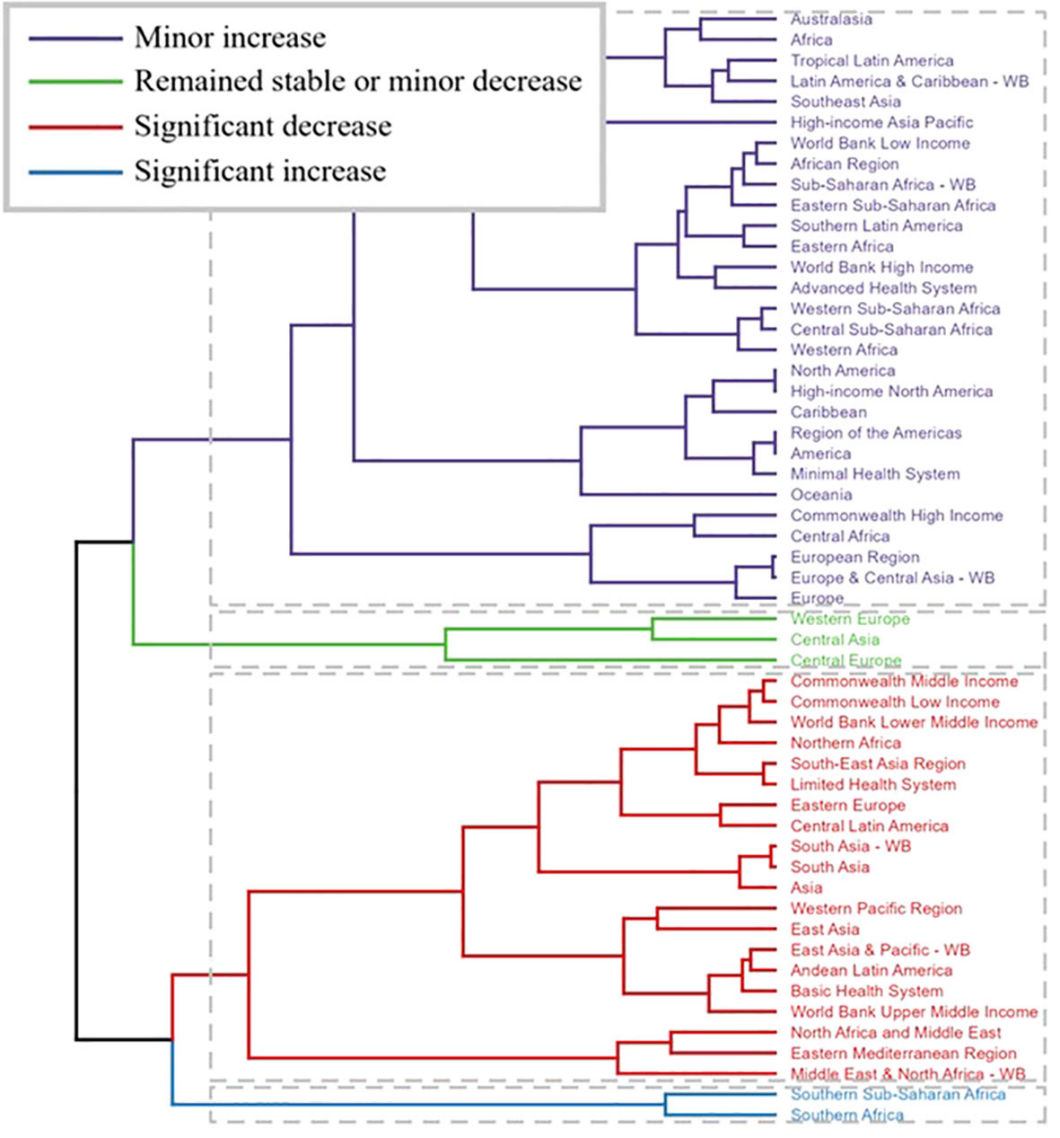
Cluster analysis.

The clustering results of thyroid cancer in the AYA (Adolescent and Young Adult) population highlight distinct global trends in disease burden and health system responses (**Figure 4**). Regions such as Australasia, Africa, Southeast Asia, and Latin America exhibit a minor increase in thyroid cancer incidence and outcomes, suggesting these regions are experiencing a modest rise in disease rates or improvements in healthcare management. Meanwhile, regions like Western Europe, Central Asia, and Central Europe have largely remained stable or experienced a minor decrease, indicating a steady or slightly declining burden. On the other hand, regions including Northern Africa, South Asia, Eastern Europe, and Commonwealth Middle Income countries demonstrate a significant decrease in outcomes, suggesting a marked decline in performance related to thyroid cancer control, possibly due to deteriorating healthcare systems or increased disease burden. However, Southern Africa and the Middle East & North Africa stand out with significant increases in their health metrics, implying notable improvements in managing thyroid cancer in the AYA population in these regions. This clustering underscores the varying responses to thyroid cancer globally, with some regions improving outcomes while others struggle with significant challenges.

### 3.5 Trend analysis (1990–2021) and predictive analysis (2022–2050)

**Figure 5:**
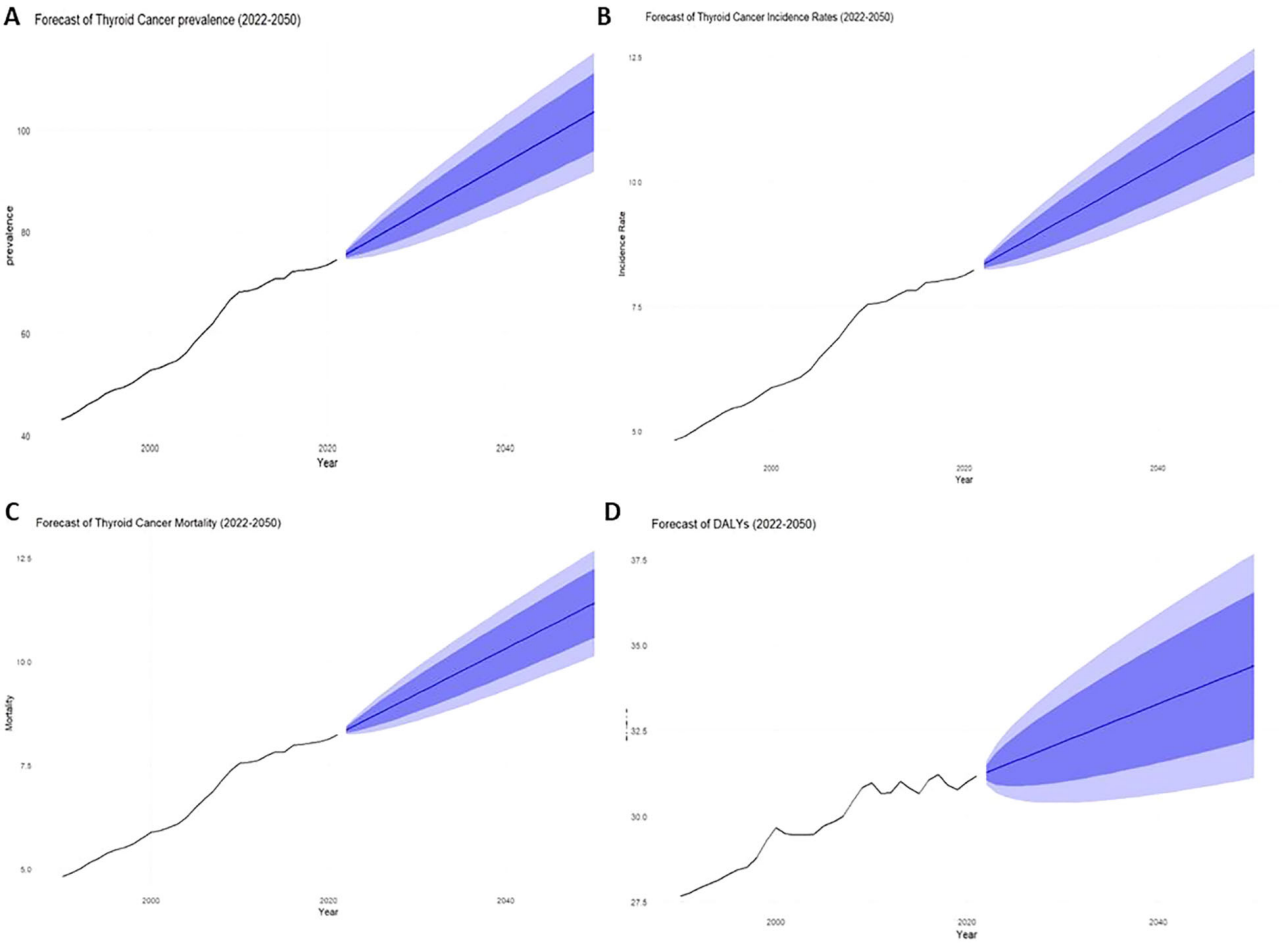
Total 1999-2021 and 2022-2050 actual and projected rates for AYA thyroid cancer patients [**(A)** Prevalence, **(B)** Incidence, **(C)** Mortality, **(D)** DALYs].

The projected trends for thyroid cancer through 2050 show significant increases across multiple indicators. The overall prevalence is projected to reach 103.62 per million (95% CI: 91.85-115.40) by 2050 (**Figure 5A**). The incidence rate is forecasted to rise to 11.41 per 100,000 (95% CI: 10.13-12.69) (**Figure 5B**), while the mortality rate, which was 0.452 in 2021, is expected to increase slightly to 0.479 (95% CI: 0.462-0.496) by 2050 (**Figure 5C**). The burden of disease, as measured by DALYs, is projected to be 34.41 per million (95% CI: 31.12-37.69) by 2050 (**Figure 5D**). These projections underscore the growing impact of thyroid cancer on public health in the coming decades.

**Figure 6:**
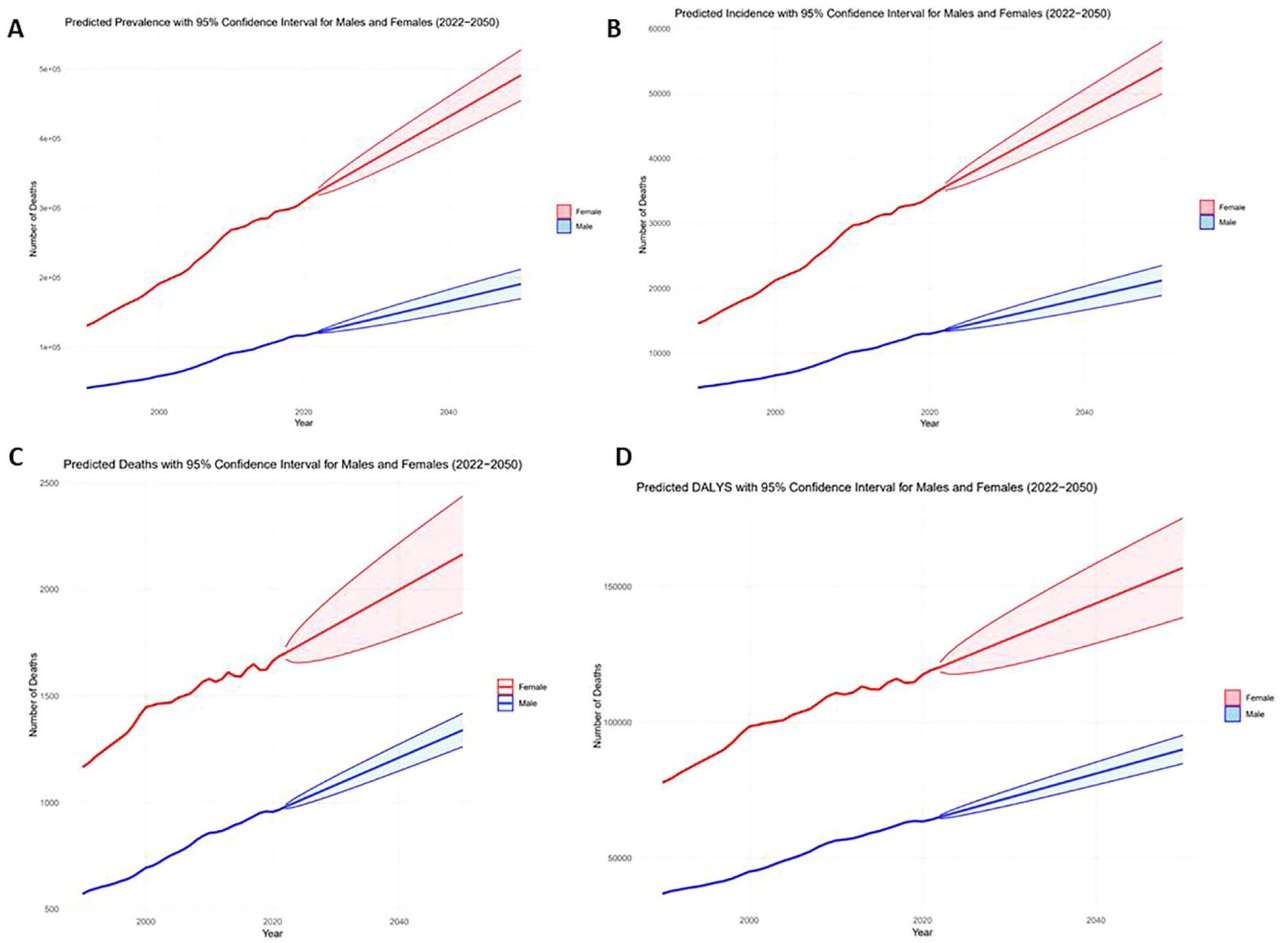
Actual 1999-2021 and projected 2022-2050 numbers in the AYA thyroid cancer population, by gender [**(A)** Prevalent cases, **(B)** Number of incidents, **(C)** Number of deaths, **(D)** DALYs].

**Figure 7:**
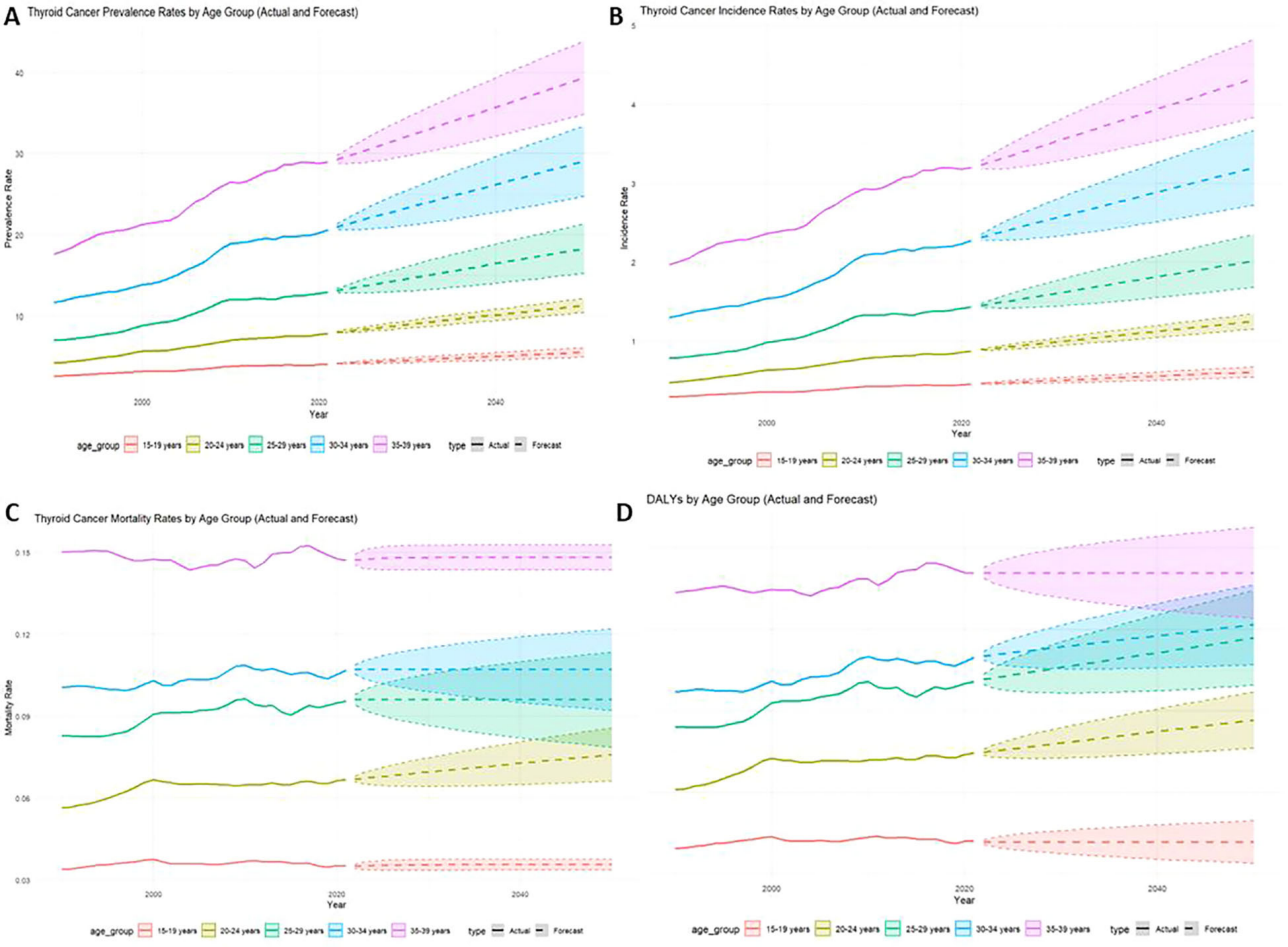
Actual rate of thyroid cancer in adolescents, 1999-2021, and predicted rate, 2022-2050, by Age group [**(A)** Prevalence, **(B)** Incidence, **(C)** Mortality, **(D)** DALYs].

**Figure 8:**
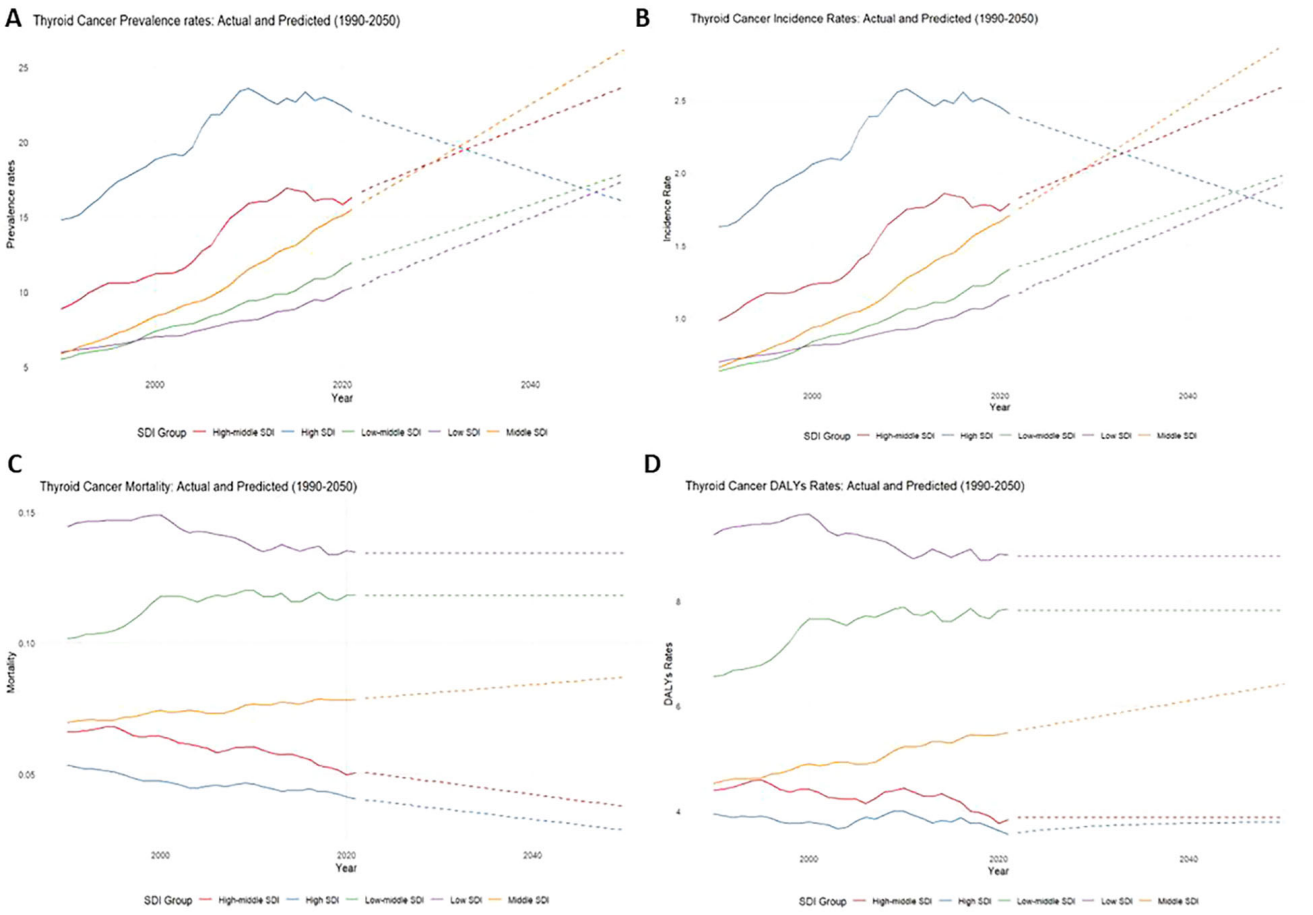
Actual rate of thyroid cancer in adolescents, 1999-2021, and predicted rate, 2022-2050, by SDI group [**(A)** Prevalence, **(B)** Incidence, **(C)** Mortality, **(D)** DALYs].

The results indicate a substantial projected increase in the thyroid cancer burden between 2022 and 2050, particularly among females, older age groups (30-39 years), and lower-SDI regions. Females are expected to account for significantly higher numbers of prevalent cases, incidents, and deaths compared to males, with a widening gap over time (**Figure 6**). Older age groups will bear the greatest burden, with the 2050 incidence rate projected to reach 4.33 (95% CI: 3.83-4.83), and the prevalence rate projected to reach 39.37 (95% CI: 34.85-43.90) (**Figures 7A, B**). Mortality rates and DALYs remain largely unchanged across most groups, although there is an upward trend in the 20-24 age group, where mortality is expected to increase from 0.067 to 0.076 per million in 2021 (95% CI: 0.066-0.086) (**Figures 7C, D**). Notably, high-SDI regions are projected to experience a decline in both incidence and prevalence, with the prevalence decreasing from 2.41 in 2021 to 1.76 in 2025, and the incidence rate declining to 17.89 per million by 2050 (**Figures 8A, B**). Additionally, the prevalence of medium-SDI regions will surpass that of medium-high SDI by 2030, with the incidence rate expected to surpass medium-high SDI in 2029 (**Figures 8A, B**). The mortality rate in high-SDI regions is also predicted to decrease from 0.0406 to 0.0285 by 2050, with a similar downward trend observed in medium-high SDI regions (**Figures 8C, D**). Overall, while the burden of thyroid cancer grows in lower-income regions, high-SDI countries see a decreasing trend, reflecting disparities in thyroid cancer dynamics across socio-demographic groups. Confidence intervals widen over time, indicating increasing uncertainty in long-term predictions.

## 4 Discussion

TC has emerged as a significant global health concern, with its age-standardised incidence rate (ASIR) rising consistently between 1990 and 2019 (23). This increase was particularly pronounced among women, who faced a higher overall burden of disease compared to men at both time points. The male-to-female ASIR ratio grew from 0.41 in 1990 to 0.51 in 2019, while the age-standardised mortality rate (ASDR) also increased, rising from 0.60 to 0.82 (23). Over recent decades, thyroid cancer has become one of the most prevalent malignancies of the endocrine system. AYA, aged 15 to 3, is especially common, with a 5-year survival rate as high as 98% (24). However, treatments such as radioactive iodine therapy and thyroid hormone suppression can lead to long-term adverse effects. Notably, AYA patients who developed synchronous or early secondary thyroid cancer faced a higher risk of death compared to those whose secondary cancers developed years later (25). In response to these trends, we conducted a comprehensive analysis of gender and temporal trends in TC in young adults and used an ARIMA model to predict the prevalence, incidence, and mortality DALYs of thyroid cancer globally and under different characteristic stratifications for the next 20 years.

Based on EAPC trends from 1990 to 2021, the EAPC for the AYA population (15-39 years) decreases with increasing age group, but the EAPC for disease, morbidity, and DALYs peaks for the 20-24 age group. This peak can be attributed to several key factors. Enhanced diagnostic sensitivity and the potential for overdiagnosis have likely played a significant role, as improved medical technologies and increased health screenings during early adulthood have led to the detection of subclinical thyroid cancers, which may not have caused symptoms or mortality otherwise (26). Additionally, exposure to environmental endocrine-disrupting chemicals (EDCs), found in everyday products like plastics and personal care items, may interfere with thyroid function, increasing cancer risk in younger populations (27). The heightened sensitivity of the thyroid gland to these external factors during early adulthood likely contributes to the rapid rise in TC incidence (28). Lifestyle factors, including rising rates of obesity and altered dietary habits, further exacerbate this trend by promoting metabolic changes that can increase cancer susceptibility (29, 30). As the AYA population ages beyond 24 years, EAPC begins to decline. This may be due to a decrease in the number of new cases diagnosed, changes in diagnostic methods, or the natural aging process that may alter risk factors. However, the overall burden in the 25-39 age group remains high, even though the relative growth rate (EAPC) becomes less steep as individuals approach their 30s, suggesting that while the incidence of new cases stabilizes, the long-term effects and health burden persist.

The substantial regional variations in the incidence and prevalence of TC among AYA populations, as revealed by the GBD database, can be attributed to several factors (27). Environmental and lifestyle differences, such as higher exposure to carcinogens, tobacco use, and dietary patterns in regions like the MENA, likely contribute to the higher rates observed. Genetic predispositions in these populations could also play a significant role in their susceptibility to TC (31). In contrast, the lower incidence in Western Sub-Saharan Africa might stem from a combination of underreporting due to limited healthcare access and fewer environmental risk factors. Additionally, different challenges, such as economic problems, armed conflicts, and political instability, alongside the uneven distribution of equipped or experienced healthcare staff, have led to unfavourable figures in some countries (32). Stigmatization of and mistrust in the healthcare system further exacerbate these challenges, limiting access to early diagnosis and treatment, thus impacting the overall disease burden. Addressing these socio-political factors could significantly improve cancer care and reporting in affected regions.

Mortality and DALY patterns also show stark contrasts across regions (33). Mortality and disability-adjusted life year (DALY) patterns also show contrasts across regions, with a downward trend in Europe (especially Central and Western Europe), which is largely attributed to improved healthcare, early diagnosis, advanced treatment options, and strong public health interventions, such as the finding of the Westland Cancer Screening Programme in Sweden that the European Thyroid Imaging Reporting and Data System (EU-TIRADS) that the selective use of fine needle aspiration cytology (FNAC) in thyroid cancer management has reduced unnecessary surgery (34). Regions such as Eastern Sub-Saharan Africa, which have the highest mortality rates and DALYs from TC, face significant challenges due to insufficient medical resources, delayed diagnoses, and a lack of effective treatment programs (35). Non-communicable diseases like cancer have now emerged as the leading cause of death in this region, exacerbating the healthcare crisis. The disproportionate burden of cancer may also be tied to rapid urbanization, coupled with unhealthy lifestyles often associated with poverty and inequality (36). These factors highlight the urgent need for targeted interventions and a more equitable allocation of resources to reduce disparities in cancer outcomes, particularly in regions with rising mortality rates and high DALY burdens.

Libya reports the highest global DALY rate, underscoring the significant disease burden in North Africa. Furthermore, Libya also exhibits one of the highest morbidity rates, with a prevalence exceeding 40 per 100,000. Despite these figures, there is a relative scarcity of reports specifically addressing thyroid-related diseases in the country, suggesting a gap in focused research or reporting on this condition (37). Despite the relatively low incidence of thyroid cancer in India, the burden of morbidity and mortality remains significant. The country recorded 815 deaths, highlighting a substantial disease impact, which is likely amplified by its large population and healthcare disparities. A key factor contributing to this burden is the stark contrast between urban and rural healthcare systems. In urban areas, access to diagnostic facilities and treatment protocols allows for more effective disease management. Conversely, rural areas suffer from limited healthcare infrastructure, leading to delayed diagnoses and inadequate treatment, which results in higher mortality rates and poorer outcomes (38). Notably, between 2006 and 2012, the incidence of thyroid cancer in Trivandrum, Kerala, surged by 93%, reaching 13.3 per 100,000 in 2012. This sharp increase is likely driven by overdiagnosis, particularly among those under 40, exacerbating the apparent disease burden (39, 40).

The incidence, prevalence, mortality and peak DALYS of thyroid cancer in the AYA female population coincide with the time when women experience major hormonal fluctuations such as puberty and pregnancy. One potential causative factor is premature oestrogen exposure, and exposure to higher levels of oestrogen during the reproductive years may increase the risk of TC (41). The onset of menstruation (the first menstrual period) and subsequent hormonal cycles may lead to increased susceptibility, particularly through oestrogen stimulation of thyroid cells. Pregnancy may further increase the risk of thyroid cancer, which is the second most common malignancy during pregnancy, after breast cancer, with 14 out of every 100,000 pregnant women having thyroid cancer (42). Nearly 10 per cent of thyroid cancer cases in women of childbearing age are diagnosed during pregnancy or early in the postpartum period (43). Elevated human chorionic gonadotropin (hCG) during normal pregnancy stimulates TSH receptors and directly increases thyroid hormone production, leading to elevated serum free T4 and decreased serum TSH during pre-pregnancy (44). TSH is a driver of thyroid cancer growth, and therefore elevated hCG and TSH receptor stimulation is considered a factor that may promote thyroid cancer growth during pregnancy (45).

Many studies explored the seasonal variations in thyroid function among women of reproductive age, with a focus on thyrotropin (TSH), free triiodothyronine (FT3), free thyroxine (FT4), and the TSH index (TSHI) (21, 46). They observed that TSH and TSHI peaked in winter and were lowest in summer, indicating a clear seasonal pattern influenced by external environmental factors such as temperature and photoperiod. These fluctuations in thyroid hormone levels may contribute to the risk of thyroid cancer, as elevated TSH during winter could stimulate thyroid cell proliferation, potentially increasing the risk of malignant transformation over time (11). The relationship between temperature and TSH levels, where colder weather leads to higher TSH, suggests that thyroid function is regulated by environmental changes, potentially causing oxidative stress and DNA damage in thyroid cells, which may contribute to cancer development (47, 48). Given the seasonality in thyroid function, clinicians should consider the timing of diagnostic evaluations and treatments for thyroid-related diseases, especially in high-risk populations. The use of ARIMA (autoregressive moving average) and exponential smoothing models in this study is justified due to their ability to effectively capture both short-term fluctuations and long-term trends in thyroid hormone levels. These models provide valuable insights into the seasonal dynamics of thyroid function and its potential impact on thyroid cancer risk, while also enabling predictions of future diabetes incidence. (49).

The rationale for using both Exponential Smoothing and ARIMA to forecast TC incidence lies in their complementary strengths for different forecasting horizons and data behaviours. Exponential Smoothing is ideal for short-term forecasts as it quickly adapts to recent trends, making it suitable for predicting incidence rates driven by current healthcare policies and detection practices (e.g., 2021 to 2025) (50). In contrast, ARIMA excels in capturing historical patterns and long-term trends, making it better suited for extended forecasts (e.g., 2026 to 2050), where broader factors such as demographic aging and healthcare improvements are more influential (51). Together, these models address both short-term fluctuations and long-term structural shifts in incidence rates. ARIMA’s ability to handle non-stationary data, common in healthcare time series, and its proven success in forecasting non-seasonal, annual datasets like the GBD data, supports its application (52). While Exponential Smoothing lacks substantial evidence in GBD contexts, its flexibility in adapting to recent changes makes it valuable for short-term forecasts, even without seasonality. Combining both models enhances predictive robustness by addressing both short-term variations and long-term trends, with convergences boosting confidence in forecasts and divergences highlighting areas for further investigation.

This study presents several strengths and limitations that warrant discussion. One of the main strengths is its focus on the adolescent and young adult (AYA) population, a group that is often underrepresented in cancer research despite having distinct patterns of thyroid cancer incidence and outcomes. This focus addresses a critical gap and provides valuable insights into thyroid cancer dynamics in this age group. Additionally, the use of advanced predictive modelling techniques, including ARIMA and Exponential Smoothing models, enhances the study by offering both short- and long-term forecasts, providing a nuanced understanding of future trends in thyroid cancer burden. However, the study also has limitations. For example, the ARIMA model generated negative predictions for the 20-24 age group, highlighting the model’s limitations in handling small and volatile datasets without non-negativity constraints. This suggests caution in interpreting predictions for this age group. Furthermore, the study did not account for lifestyle risk factors or exposure to environmental toxins, such as smoking or endocrine-disrupting chemicals, which are known to influence thyroid cancer risk. This omission may limit the comprehensiveness of the analysis, as these factors play a crucial role in shaping thyroid cancer outcomes in different populations.

## Data Availability

The original contributions presented in the study are included in the article/supplementary material. Further inquiries can be directed to the corresponding authors.
